# Outcome of contralateral C7 transfers to different recipient nerves after global brachial plexus avulsion

**DOI:** 10.1002/brb3.1174

**Published:** 2018-11-22

**Authors:** Yuzhou Liu, Xun Yang, Kaiming Gao, Hu Yu, Feng Xiao, Yongqing Zhuang, Jie Lao

**Affiliations:** ^1^ Department of Hand Surgery, Huashan Hospital Fudan University Shanghai China; ^2^ Key Laboratory of Hand Reconstruction Ministry of Health Shanghai China; ^3^ Shanghai Key Laboratory of Peripheral Nerve and Microsurgery Shanghai China; ^4^ Department of Hand surgery, Jing’an District Center Hospital Fudan University Shanghai China; ^5^ Hand and Microvascular Surgery Department Shenzhen People’s Hospital Shenzhen, Guangdong Province China

**Keywords:** biceps branch, brachial plexus, contralateral C7, median nerve, triceps branch

## Abstract

**Introduction:**

Contralateral cervical seventh nerve root (CC7) transfer has been widely applied for treatment of traumatic brachial plexus injury. The purpose of the study was to evaluate outcomes of patients with global brachial plexus avulsion (GBPA) after CC7 transfer and compare the recoveries of median nerve as the only recipient nerve and one of the multiple recipient nerves.

**Methods:**

A retrospective review of 51 patients treated with CC7 transfers after GBPA was carried out. The British Medical Research Council (MRC) grading system and range of joint motion (ROM) were used for motor and sensory assessment.

**Results:**

The effective rates of FCR were 57.7%, 45.5%, and 36.4% in CC7 transfer to median nerve (CC7‐Md), CC7 transfer to median nerve and biceps branch (CC7‐Md+Bic) and CC7 transfer to median nerve and triceps branch (CC7‐Md+Tric) groups, respectively. There were no statistical differences no matter in FCR or FDS among groups. The effective rate in biceps had no significant difference with that in triceps. The effective sensory recovery rate was 65.4%, 54.5%, and 36.4% in CC7‐Md, CC7‐Md+Bic, and CC7‐Md+Tric groups. There were no statistical differences in the sensory effective recovery rate among groups. All the ROMs were improved significantly after surgery. The improvement of ROM of elbow flexion after surgery in CC7‐Md+Bic group was significantly larger than that of elbow extension after surgery in CC7‐Md+Tric group (*p* = 0.047).

**Conclusions:**

The CC7 transfer contributed to the functional improvement of the hand and wrist for the patients with global brachial plexus avulsion. The whole CC7 could be used to repair more than one recipient nerve (including median nerve) without affecting the recovery of median nerve. When CC7 was used to repair two nerves, biceps branch might be preferred to choose as one recipient nerve rather than triceps branch.

## 1 INTRODUCTION

Traumatic brachial plexopathies can be devastating injuries (Alnot, 1995; Dubuisson & Kline, 2002; Kim, Cho, Tiel, & Kline, 2003; Kline & Hudson, 1995; Midha, 1997), and global root avulsion remains a major reconstructive challenge (Chuang & Hernon, 2012). Widely used nerve transfer sources for global brachial plexus injuries include the intercostals (Chuang, 1995; Minami & Ishii, 1987; Tomita, Tsai, Burns, Karaoguz, & Ogden, 1983; Tsuyama and Hara, 1973), spinal accessory (Allieu & Cenac, 1988; Samardzic, Grujicic, Antunovic, & Joksimovic, 1990; Songcharoen, Mahaisavariya, & Chotigavanich, 1996), phrenic nerve (Gu et al., 1989), and contralateral cervical seventh nerve root (CC7) (Gu et al., 1991). In 1986, Gu designed CC7 as a donor nerve to repair the injured nerves on the opposite side for the treatment of traumatic brachial plexus avulsion (Gu et al., 1991). In Wang et al. (2013) reported on CC7 nerve transfer with direct coaptation to restore lower trunk function after traumatic brachial plexus avulsion. There were satisfactory recoveries of finger flexion and wrist flexion in this series. CC7 transfer can provide finger sensation in the paralyzed hand and restore motor function of the shoulder, elbow, or hand, which has been widely applied for treatment of traumatic brachial plexus injury (Yang, Chang, & Chung, 2015). In the present study, we analyzed the results of 51 patients treated with CC7 transfer to different recipient nerves after global brachial plexus avulsion (GBPA).

## 2 METHODS

A retrospective review of 51 patients treated with CC7 nerve transfer after posttraumatic global brachial plexus injury was carried out. The clinical research was reviewed and approved by the institutional review board of Huashan Hospital Affiliated to Fudan University (Approval No: 2015–163), and all patients gave informed consent. The inclusion criteria included global root avulsion (C5 to T1 avulsion) and CC7 root as donor nerve in the treatment. The exclusion criteria included diabetes, Volkmann contracture, fracture on the affected limb, and brain trauma. According to medical records, inclusion and exclusion criterions, the enrolled patients were confirmed. Then the patients were called in our department for outcome measure.

All 51 patients were confirmed to have global root avulsion by surgical exploration. All the patients were treated between 2006 and 2014. There were 49 males and 2 females. Motorcycle accidents accounted for injuries in 38 patients. Other road accidents included a pedestrian, a motor vehicle, and two bicycle accidents. Four patients had traction injury of upper limb and four patients suffered weight dropping on the shoulder. Explosion led to a patient's brachial plexus avulsion. The average follow‐up period was 4.2 years. The average age at the time of injury was 26.5 years, but the range was 13–59 years. We removed the extreme age range patients (13 and 59 years old) from the statistics. The average delay to surgery was 2.7 months, but the highest is 17 months. We removed the long preoperative delay case (17 months) from study to get better comparison between different series (Table 1). Therefore, 48 patients were involved in the statistical analysis. All the operations were done by the same group of surgeons.

**Table 1:** A total of 51 patients with global root avulsion brachial plexus injuries

Male	49
Female	2
Age of injury (years)	13–59 (mean 26.5), the extreme age range patients (13 and 59 years old) were removed.
Follow‐up period (years)	3–11(mean 4.2)
Delay to OR (months)	1–17 (mean 2.7), the long preoperative delay case (17 months) was removed.
Cause	
Motorcycle accident	38
Pedestrian accident	1
Bicycle accident	2
Motor vehicle accident	1
Traction injury by a machine	4
Weight dropping on the shoulder	4
Explosion	1
48 patients were involved in the statistical analysis.	
Recipient nerve	
Median nerve	26
Median nerve +biceps branch	11
Median nerve +triceps branch	11

OR, operation.

### 2.1 Surgical technique

A transverse incision was made superior to the clavicle on the contralateral side for exploring C7 root. C7 nerve root was confirmed by anatomic identification of its location and electric stimulation, which resulted in shoulder adduction, elbow extension, and wrist extension (Gu, Xu, Chen, Wang, & Hu, 2002). CC7 root was blocked by 2% lidocaine epineurium injection.

**Figure 1:**
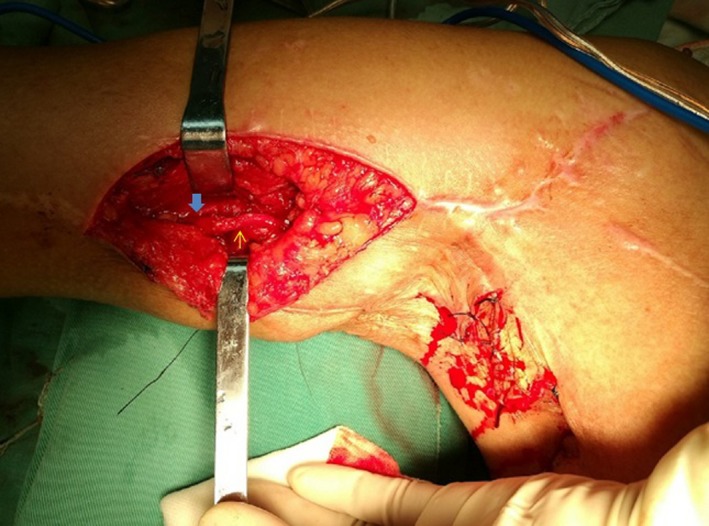
CC7 stage II: The ulnar nerve was sutured to the median nerve (fine arrow‐ulnar nerve, thick arrow‐median nerve)

When the recipient nerve was median nerve, triceps branch, or biceps branch, the vascularized ulnar nerve was adopted as nerve graft. The first stage: The vascularized ulnar nerve graft based on the superior ulnar collateral artery was harvested from the affected arm and passed across the chest through a subcutaneous tunnel to the normal neck from the opposite axilla (Gao, Lao, Zhao, & Gu, 2013). Then the ulnar nerve was sutured to the whole CC7 nerve root under 2.5 × magnification, using 8–0 microsutures. The second stage (4–6 months after the first stage): The ulnar nerve on the affected side was resected and sutured to the recipient nerve (Figure 1). The patient was immobilized by a head and arm rack to keep the head from turning to the affected side for one month.

### 2.2 Postoperative rehabilitation

Physical therapy and electrostimulation therapy were started 4 weeks postoperatively. Patients were instructed to adduct his contralateral shoulder against resistance, while doing the action of the affected limb according to the recipient nerve. For example, if the recipient nerve was median nerve, the patient was instructed to practice wrist and finger flexion in the affected limb while adducting his contralateral shoulder against resistance. We formulated a scheme for patients: Physical therapy was done three times per day and each time physical therapy lasted for 1 hr.

The electrostimulation therapy was carried out twice per day. The postoperative rehabilitation, including physical therapy and electrostimulation therapy, lasted for at least 2 years.

### 2.3 Evaluation

The British Medical Research Council (MRC) grading system (Medical Research Council, 1976) and range of joint motion (ROM) were used for motor and sensory assessment. Return of muscle power of M3 or better was regarded as effective. S3 or better indicated an effective sensory recovery (Liu, Lao, Gao, Gu, & Zhao, 2013).

The satisfaction with surgery was shown by the following question: “If you were to go back in time, would you choose to have the contralateral C7 nerve transfer again?” with the following possible responses: (a) definitely yes, (b) probably yes, (c) uncertain, and (d) definitely not (Ahmed‐Labib, Golan, & Jacques, 2007).

### 2.4 Statistical analysis

All analyses were performed using Statistical Package for Social Sciences (SPSS version 19.0, Chicago, IL, USA). Comparison between preoperative and postoperative ROM was analyzed using *t* test for parametric data and Wilcoxon signed rank sum test for nonparametric data. P‐values were two‐tailed, and *p* values <0.05 were considered significant.

## 3 RESULTS

**Figure 2:**
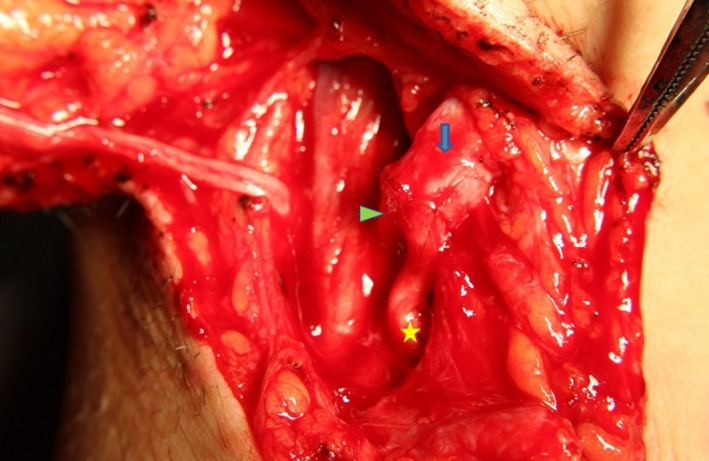
CC7 stage II: The ulnar nerve was sutured to the median nerve and biceps branch (triangle‐median nerve, thick arrow‐ulnar nerve, star‐biceps branch)

According to the difference of recipient nerves, the patients could be divided into three groups. 26 patients had CC7 transfer to median nerve (CC7‐Md). 11 patients had CC7 transfer to median nerve and biceps branch (CC7‐Md+Bic; Figure 2), while 11 patients had CC7 transfer to median nerve and triceps branch (CC7‐Md+Tric).

### 3.1 MRC grading (motor power)

**Figure 3:**
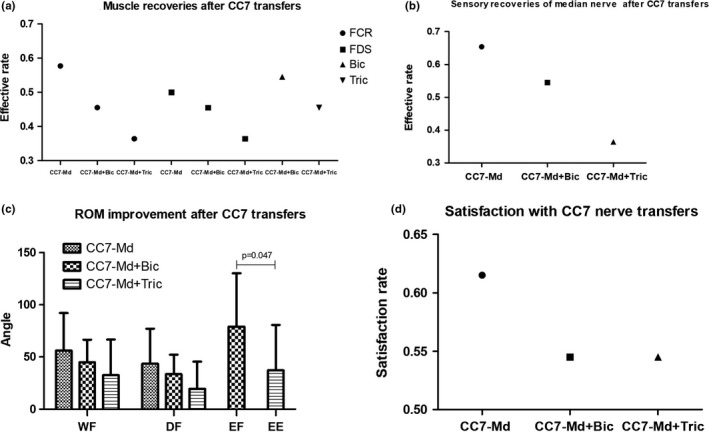
(a) Muscle power recoveries after CC7 transfer to different recipient nerves. (b) Sensory recoveries of median nerve after CC7 transfer to different recipient nerves. (c) ROM improvement after CC7 transfer to different recipient nerves. (d) Satisfaction with CC7 transfer to different recipient nerves

All of the muscle strength was M0 in the affected limb with global brachial plexus avulsion preoperatively. The muscles tested were the main targets of the recipient nerves. Median nerve: flexor carpi radialis (FCR) and flexor digitorum superficial (FDS); Biceps branch: Biceps; Triceps branch: Triceps. As Figure 3a shown, the effective rates of FCR were 57.7%, 45.5%, and 36.4% in CC7‐Md, CC7‐Md+Bic, and CC7‐Md+Tric groups, respectively. The effective rates of FDS were 50.0%, 45.5%, and 36.4% in CC7‐Md, CC7‐Md+Bic, and CC7‐Md+Tric groups, respectively. In CC7‐Md+Bic group, 54.5% of patients got M3 or M4 in biceps. About 54.5% of patients got less than M3 in triceps in CC7‐Md+Tric group. All the grades of abductor pollicis brevis (APB) after different CC7 nerve transfers were <M3. Comparing muscle power recoveries of median nerve after whole CC7 transfer to different recipient nerves, there were no statistical differences no matter in FCR or FDS. The effective rate in biceps had no significant difference with that in triceps (Table 2).

**Table 2:** Muscle recoveries after CC7 transfer to different recipient nerves

Type	M0	M1	M2	M3	M4	Effective rate (%)	*p*
CC7‐Md(FCR)	2	4	5	12	3	57.7	0.719 (Md:Md+Bic)
CC7‐Md+Bic(FCR)	0	2	4	4	1	45.5	1.000 (Md+Bic:Md+Tric)
CC7‐Md+Tric(FCR)	4	1	2	2	2	36.4	0.295 (Md+Tric:Md)
CC7‐Md(FDS)	4	5	4	11	2	50.0	1.000 (Md:Md+Bic)
CC7‐Md+Bic(FDS)	0	1	5	5	0	45.5	1.000 (Md+Bic:Md+Tric)
CC7‐Md+Tric(FDS)	4	1	2	4	0	36.4	0.495 (Md+Tric:Md)
CC7‐Md+Bic(Bic)	1	1	2	2	5	54.5	0.670 (Md+Bic:Md+Tric)
CC7‐Md+Tric(Tric)	1	5	0	2	3	45.5	

Note

FCR, flexor carpi radialis; FDS, flexor digitorum superficial; CC7, Contralateral cervical seventh nerve root.

Md:Md+Bic:comparison of the effective rates between CC7‐Md and CC7‐Md+Bic groups.

Md+Bic:Md+Tric: comparison of the effective rates between CC7‐Md+Bic and CC7‐Md+Tric groups.

Md+Tric:Md: comparison of the effective rates between CC7‐Md+Tric and CC7‐Md groups.

### 3.2 MRC grading (sensory assessment)

The radial side of palm and the palm‐sides of thumb, index, and middle fingers were the regions which median nerve dominated. Figure 3b showed different sensory recoveries of median nerve according to different recipient nerves. In CC7‐Md group, there were three patients with S4 recovery, 14 patients with S3, 2 patients with S2, and 7 patients with S0. The effective sensory recovery rate was 65.4%. The sensation of the radial side of palm and the 1–3 palmar digit recovered to S3 in 6 patients, S1 in two patients, and S0 in three patients in CC7‐Md+Bic group, which indicated the effective sensory recovery was 54.5%. In CC7‐Md+Tric group, there were one patient with S4 recovery, three patients with S3, one patient with S2, three patients with S1 and three patients with S0. The total effective rate of median nerve sensory recovery after different CC7 nerve transfer was 56.3%. Comparing sensory recoveries of median nerve after whole CC7 transfer to different recipient nerves, there were no statistical differences in the sensory effective recovery rate (Figure 3b).

### 3.3 ROM improvement

The wrist and digital flexion ranges were all improved significantly in CC7‐Md, CC7‐Md+Bic, and CC7‐Md+Tric groups (*p* < 0.05). The total EF (elbow flexion range) and EE (elbow extension range) were also significantly improved in CC7‐Md+Bic and CC7‐Md+Tric groups, respectively, compared with those before surgery. Figure 3c showed ROM improvement after CC7 transfer to different recipient nerves. The ROMs of WF and DF both decreased from CC7‐Md group to CC7‐Md+Tric group via CC7‐Md+Bic group, but there were no statistical differences among groups. The ROM of EF in CC7‐Md+Bic group was significantly larger than that of EE in CC7‐Md+Tric group (*p* = 0.047).

### 3.4 Satisfaction with surgery

In the CC7 transfer to median nerve group, 16 patients answered “definitely yes” or “probably yes” in response to the question on their readiness to undergo surgery again. Ten patients answered “uncertain”. The satisfaction rate was 61.5% in the CC7‐Md group. The satisfaction rates of the CC7‐Md+Bic and CC7‐Md+Tric transfer were both 54.5% (Figure 3d). There were no significant differences of the satisfaction with surgery between groups. The total satisfaction rate for all patients with CC7 nerve transfer was 58.3%.

### 3.5 Complications

A total of 38 patients experienced paresthesia on the thumb, index, and middle pulp of the donor hand within three months after surgery and the sensory deficit completely recovered spontaneously in all patients now.

## 4 DISCUSSION

This retrospective study evaluated the functional outcomes of different CC7 nerve transfers for repairing different recipient nerves in aspects of motor strength, sensory recovery, ROM improvement, and satisfaction with surgery. The median nerve is the main recipient nerve in CC7 nerve transfers. The motor and sensory effective recovery rates of median nerve approached around 50%, and the wrist and digital flexion ranges were improved significantly by CC7 transfer to median nerve in the study. Whole C7 contains 27,000–41,000 nerve fibers and median nerve contains 18,288 nerve fibers (Bonnel & Rabischong, 1980). The difference of the numbers of nerve fibers between whole CC7 and median nerve explained the study result “the effect recovery rates of FCR and FDS after whole CC7 only transfer to median nerve had no statistical differences with those after whole CC7 transfer to median nerve and other nerves.” According to the result of ROM improvement, the wrist and digital flexion ranges were improved significantly by CC7 transfer to median nerve, which implied CC7 nerve transfer contributed to the functional improvement of the hand and wrist for the patients with global brachial plexus avulsion. The effective rate of biceps was higher than that of triceps and the ROM of EF in CC7‐Md+Bic group was significant larger than that of EE in CC7‐Md+Tric group, which indicated the recovery of biceps was better than that of triceps after CC7 transfers. The satisfaction result showed more than half of the patients were basically satisfied with CC7 nerve transfer. The patients’ subjective evaluation reflected CC7 nerve transfer was an acceptable operation by most of the people.

In August 1986, the world's first case of contralateral C7 nerve transfer was finished by Gu et al. (1992) and he reported the overall motor recovery rate (≥M3) was 50%–80% depending on different recipient nerves and the sensory recovery rate (≥S3) was above 60% (Zhang & Gu, 2011). Waikakul (Waikakul, Orapin, & Vanadurongwan, 1999) reported that only 52% of patients had ≥M3 recovery after contralateral C7 transfer to musculocutaneous nerve, and 20% recovery for the extensor of wrist/finger and 29% recovery for finger flexor. David Chwei‐Chin Chuang, et al. (Chuang & Hernon, 2012) had a minimum 4‐year follow‐up on Contralateral C7 nerve transfers for brachial plexus injuries. The success rates of finger flexion strength were 55% and 39% for CC7‐Md and CC7‐Md+Bic groups, respectively. The success rate for recovery of elbow flexion in CC7‐Md+Bic group was 83%. Terzis reported the summing rates of fair (M2+~M3), good (M3+~M4−), and excellent (M4+~M5−) in 56 cases were 74% for biceps; 57% for triceps; 62% for wrist and finger flexors; and 50% for wrist and finger extensors, respectively (Terzis & Kokkalis, 2009). Compared with the results reported previously, our results showed that 57.7% of 26 patients had ≥M3 recovery for FCR, 50.0% had recovery for FDS, and 65.4% of 26 patients had ≥S3 recovery after CC7‐Md, which was approximated to Gu's reports. Gao, Lao, Zhao, and Gu (2013) reported the outcome of CC7 transfer to two recipient nerves in 22 patients with GBPA. About 68.2% of patients achieved the motor recovery of wrist and finger flexor to M3 or greater, and 45.5% of patients got the sensory recovery of median nerve to S3 or greater. In his study, there was no comparison of the median nerve recovery between CC7‐Md and CC7‐Md+other nerve. Wang et al. (2013) reported 75 patients had CC7 transfer to lower trunk and musculocutaneous nerve. Motor function with M3+ or greater was attained in 60% of the patients for elbow flexion, 64% of the patients for finger flexion, 53% of the patients for thumb flexion, and 72% of the patients for wrist flexion. The results proved median nerve and biceps branch reinnervated by cC7 achieved satisfactory recovery, which coincided with our conclusion.

The recovery of intrinsic muscle after CC7 nerve transfer was still a problem. In this study, all the grades of APB muscle after different CC7 nerve transfers were <M3, including eight patients with M1, one patient with M2, and the other with M0. The main reason was still a long time for nerve fibers growing from the donor nerve (CC7) to the intrinsic muscle and a faster speed of intrinsic muscle atrophy than nerve growing. Another reason was that the higher number of nerve fibers from C7 root was transmitted by a long ulnar nerve graft with smaller number of nerve fibers, which affected the results and long time for recovery. In the study, the patients recovered wrist and finger flexion in the affected limb while adducting his contralateral shoulder, which meant contralateral coactivation could initiate the intended movement. This was a process for motor cortical remodeling. The principle of the exercise method was synchronicity, which interferes with the utility of the recovered muscle functions. In our clinical experience, there were patients who could initiate the movement without any contralateral co‐activation after CC7 transfer. As for the sensory perception of the reinnervated hand, the stimulus was noted both in the hands of the injured side and donor side in some patients. A portion of patients felt the stimulus only in the injured side. The phenomenon might be related to sensory cortex remodeling.

This study had some limitations. Motor strength and ROM could also be improved by postoperative rehabilitation including physical therapy and electrostimulation therapy, which was an influence factor of muscle recovery. In this study, although we formulated a scheme of postoperative rehabilitation for patients, we did not collect the actual information of patients’ postoperative rehabilitation, which might induce some potential bias. The evaluation methods were MRC and ROM without electromyogram (EMG) in our study. The amplitude and latency of compound muscle action potential (CMAP) of the muscles were not used for evaluation, which were more accurate assessments of the recovery of muscle reinnervation. There was no functional outcome evaluation such as DASH questionnaire scoring to analyze the results in terms of the usefulness of the regained movements in the study. This study belonged to a single‐center clinical study, so the results had certain regional limitations.

Our study was a retrospective study. The findings of the study applied to the patients with global brachial plexus avulsion. Contralateral C7 transfer could be used to repair different recipient nerves according to the function which the patient needs to restore.

## 5 CONCLUSIONS

The CC7 nerve transfer contributed to the functional improvement of the hand and wrist for the patients with global brachial plexus avulsion. The whole CC7 could be used to repair two recipient nerves (including median nerve) without influencing on the recovery of median nerve. When CC7 was used to repair two nerves, biceps branch might be preferred to choose as one recipient nerve rather than triceps branch.

## CONFLICT OF INTEREST

I, Jie Lao, M.D, Department of Hand surgery, Huashan Hospital, Fudan University, Shanghai, China, declare that we have no conflict of interest. We have no proprietary, financial, professional, or other personal interest of any nature or kind in any product, service, or company that could be construed as influencing the position presented in, or the review of, the manuscript entitled, “Outcome of contralateral C7 transfers to different recipient nerves after global brachial plexus avulsion in 51 patients”.

## References

[brb31174-bib-0001] Ahmed‐Labib, M. , Golan, J. D. , & Jacques, L. (2007). Functional outcome of brachial plexus reconstruction after trauma. Neurosurgery, 61, 1016–1023. 10.1227/01.neu.0000303197.87672.31 18091278

[brb31174-bib-0002] Allieu, Y. , & Cenac, P. (1988). Neurotization via the spinal accessory nerve in complete paralysis due to multiple avulsion injuries of the brachial plexus. Clinical Orthopaedics and Related Research, 237, 67–74. 10.1097/00003086-198812000-00011 3191642

[brb31174-bib-0003] Alnot, J. Y. (1995). Traumatic brachial plexus lesions in the adult: Indications and results. Microsurgery, 16, 22–29. 10.1002/micr.1920160108 7658963

[brb31174-bib-0004] Bonnel, D. F. , & Rabischong, P. (1980). Anatomie et systematization du plexus brachial de I’ adulte. Anatomia Clinica 2(3), 289–298.

[brb31174-bib-0005] Chuang, D. C. (1995). Functioning free muscle transplantation for brachial plexus injury. Clinical Orthopaedics and Related Research, 314, 104–111. 10.1097/00003086-199505000-00014 7634622

[brb31174-bib-0007] Chuang, D. C. , & Hernon, C. (2012). Minimum 4‐Year follow‐up on contralateral C7 nerve transfers for brachial plexus injuries. The Journal of Hand Surgery, 37(A), 270–276. 10.1016/j.jhsa.2011.10.014 22173004

[brb31174-bib-0009] Dubuisson, A. S. , & Kline, D. G. (2002). Brachial plexus injury: A survey of 100 consecutive cases from a single service. Neurosurgery, 51, 673–683. 10.1097/00006123-200209000-00011 12188945

[brb31174-bib-0010] Gao, K. M. , Lao, J. , Zhao, X. , & Gu, Y. D. (2013). Outcome of contralateral C7 nerve transferring to median nerve. Chinese Medical Journal, 126(20), 3865–3868. 24157147

[brb31174-bib-0011] Gao, K. , Lao, J. , Zhao, X. , & Gu, Y. (2013). Outcome of contralateral C7 transfer to two recipient nerves in 22 patients with the total brachial plexus avulsion injury. Microsurgery, 33(8), 605–611. 10.1002/micr.22137 23908144

[brb31174-bib-0012] Gu, Y. D. , Wu, M. M. , Zhen, Y. L. , Zhao, J. A. , Zhang, G. M. , Chen, D. S. , … Cheng X. M. (1989). Phrenic nerve transfer for brachial plexus neurotization. Microsurgery, 10, 287–289. 2593799 10.1002/micr.1920100407

[brb31174-bib-0013] Gu, Y. , Xu, J. , Chen, L. , Wang, H. , & Hu, S. (2002). Long term outcome of contralateral C7 transfer: A report of 32 cases. Chinese Medical Journal, 115(6), 866–868. 12123554

[brb31174-bib-0014] Gu, Y. D. , Zhang, G. M. , Chen, D. S. , Cheng, X. M. , Zhang, L. Y. , Yan, J. G. , … Shen L. Y. (1991). Cervical nerve root transfer from contralateral normal side for treatment of brachial plexus root avulsions. Chinese Medical Journal, 104(104), 208–211. 2065531

[brb31174-bib-0015] Gu, Y. D. , Zhang, G. M. , Chen, D. S. , Yan, J. G. , Cheng, X. M. , & Chen, L. (1992). Seventh cervical nerve root transfer from the contralateral healthy side for treatment of brachial plexus root avulsion. Journal of Hand Surgery, 17(5), 518–521. 10.1016/S0266-7681(05)80235-9 1479244

[brb31174-bib-0016] Kim, D. H. , Cho, Y. J. , Tiel, R. L. , & Kline, D. G. (2003). Outcomes of surgery in 1019 brachial plexus lesions treated at Louisiana State University Health Sciences Center. Journal of Neurosurgery, 98, 1005–1016. 10.3171/jns.2003.98.5.1005 12744360

[brb31174-bib-0017] Kline, D. G. , & Hudson, A. R. (1995). Nerve injuries: Operative results for major injuries, entrapments, and tumors (pp. 345–548). Philadelphia, PA: WB Saunders.

[brb31174-bib-0018] Liu, Y. , Lao, J. , Gao, K. , Gu, Y. , & Zhao, X. (2013). Functional outcome of nerve transfers for traumatic global brachial plexus avulsion. Injury, 44(5), 655–660. 10.1016/j.injury.2012.02.006 22409992

[brb31174-bib-0019] Medical Research Council (1976). Aids to examination of the peripheral nervous system. Memorandum no. 45. London: Her Majesty’s Stationary Office.

[brb31174-bib-0020] Midha, R. (1997). Epidemiology of brachial plexus injuries in a multitrauma population. Neurosurgery, 40, 1182–1189. 10.1097/00006123-199706000-00014 9179891

[brb31174-bib-0021] Minami, M. , & Ishii, S. (1987). Satisfactory elbow flexion in complete (preganglionic)brachial plexus injuries: Produced by suture of third and fourth intercostals nerves to musculocutaneous nerve. Journal of Hand Surgery. American Volume, 12, 1114–1118. 10.1016/s0363-5023(87)80128-4 3320178

[brb31174-bib-0022] Samardzic, M. , Grujicic, D. , Antunovic, V. , & Joksimovic, M. (1990). Reinnervation of avulsed brachial plexus using the spinal accessory nerve. Surgical Neurology, 33, 7–11. 10.1016/0090-3019(90)90216-C 2154041

[brb31174-bib-0023] Songcharoen, P. , Mahaisavariya, B. , & Chotigavanich, C. (1996). Spinal accessory neurotizationfor restoration of elbow flexion in avulsion injuries of the brachial plexus. Journal of Hand Surgery. American Volume, 21(A), 387–390. 10.1016/S0363-5023(96)80349-2 8724466

[brb31174-bib-0025] Terzis, J. K. , & Kokkalis, Z. T. (2009). Selective contralateral C7 transfer in posttraumatic brachial plexus injuries: A report of 56 cases. Plastic and Reconstructive Surgery, 123(3), 927–938. 10.1097/PRS.0b013e31819ba48a 19319057

[brb31174-bib-0026] Tomita, Y. , Tsai, T. M. , Burns, J. T. , Karaoguz, A. , & Ogden, L. L. (1983). Intercostal nerve transfering brachial plexus injuries: An experimental study. Microsurgery, 4, 95–104. 6669011 10.1002/micr.1920040206

[brb31174-bib-0027] Tsuyama, N. , & Hara, T. (1973). Intercostal nerve transfer in the treatment of brachial plexus injury of root avulsion type In Delchef J., de Marneffe R., & Vander Elst E. (Eds). Orthopaedic surgery and traumatology, International Congress Series No. 291 (pp. 351–353). Amsterdam: Excerpta Medica.

[brb31174-bib-0028] Waikakul, S. , Orapin, S. , & Vanadurongwan, V. (1999). Clinical results of contralateral C7 root neurotization to the median nerve in brachial plexus injuries with total root avulsions. Journal of Hand Surgery, 24(5), 556–560. 10.1054/JHSB.1999.0264 10597931

[brb31174-bib-0029] Wang, S. F. , Li, P. C. , Xue, Y. H. , Yiu, H. W. , Li, Y. C. , & Wang, H. H. (2013). Contralateral C7 nerve transfer with direct coaptation to restore lower trunk function after traumatic brachial plexus avulsion. Journal of Bone & Joint Surgery – American, 95(9), 821–827. 10.2106/JBJS.L.00039 23636189

[brb31174-bib-0030] Yang, G. , Chang, K. W. C. , & Chung, K. C. (2015). A systematic review of outcomes of contralateral C7 for the treatment of traumatic brachial plexus injury: Part 2‐Donor‐site morbidity of contralateral C7 transfer for traumatic brachial plexus injury. Plastic and Reconstructive Surgery, 136(4), 480e–489e. 10.1097/PRS.0000000000001616 PMC4795155 26397267

[brb31174-bib-0031] Zhang, C. G. , & Gu, Y. D. (2011). Contralateral C7 nerve transfer—Our experiences over past 25 years. Journal of Brachial Plexus and Peripheral Nerve Injury, 6, 10. 22112443 10.1186/1749-7221-6-10 PMC3259086

